# Analyses of mutational patterns induced by formaldehyde and acetaldehyde reveal similarity to a common mutational signature

**DOI:** 10.1093/g3journal/jkac238

**Published:** 2022-09-08

**Authors:** Mahanish J Thapa, Reena M Fabros, Salma Alasmar, Kin Chan

**Affiliations:** Department of Biochemistry, Microbiology and Immunology, University of Ottawa, Ottawa, ON K1H 8M5, Canada; Ottawa Institute of Systems Biology, University of Ottawa, Ottawa, ON K1H 8M5, Canada; Department of Biochemistry, Microbiology and Immunology, University of Ottawa, Ottawa, ON K1H 8M5, Canada; Ottawa Institute of Systems Biology, University of Ottawa, Ottawa, ON K1H 8M5, Canada; Biopharmaceutical Sciences Undergraduate Program, University of Ottawa, Ottawa, ON K1N 6N5, Canada; Department of Biochemistry, Microbiology and Immunology, University of Ottawa, Ottawa, ON K1H 8M5, Canada; Ottawa Institute of Systems Biology, University of Ottawa, Ottawa, ON K1H 8M5, Canada

**Keywords:** mutagenesis, formaldehyde, acetaldehyde, genome instability, mutational pattern, mutational signature

## Abstract

Formaldehyde and acetaldehyde are reactive small molecules produced endogenously in cells as well as being environmental contaminants. Both of these small aldehydes are classified as human carcinogens, since they are known to damage DNA and exposure is linked to cancer incidence. However, the mutagenic properties of formaldehyde and acetaldehyde remain incompletely understood, at least in part because they are relatively weak mutagens. Here, we use a highly sensitive yeast genetic reporter system featuring controlled generation of long single-stranded DNA regions to show that both small aldehydes induced mutational patterns characterized by predominantly C/G → A/T, C/G → T/A, and T/A → C/G substitutions, each in similar proportions. We observed an excess of C/G → A/T transversions when compared to mock-treated controls. Many of these C/G → A/T transversions occurred at TC/GA motifs. Interestingly, the formaldehyde mutational pattern resembles single base substitution signature 40 from the Catalog of Somatic Mutations in Cancer. Single base substitution signature 40 is a mutational signature of unknown etiology. We also noted that acetaldehyde treatment caused an excess of deletion events longer than 4 bases while formaldehyde did not. This latter result could be another distinguishing feature between the mutational patterns of these simple aldehydes. These findings shed new light on the characteristics of 2 important, commonly occurring mutagens.

## Introduction

Genomic DNA is constantly damaged by intracellular processes (De Bont and van Larebeke 2004) and exposure to exogenous damaging agents (Irigaray and Belpomme 2010; Ikehata and Ono 2011; Keszenman *et al.* 2015). There are many different types of DNA damage. Intracellular DNA damaging processes include, for example oxidation of nitrogenous bases (Ames *et al.* 1993; Helbock *et al.* 1998); glycosidic bond breakage, which releases a nitrogenous base from its deoxyribose sugar (Lindahl and Nyberg 1972; Lindahl 1977; Tice and Setlow 1985; Lindahl 1993; Nakamura *et al.* 1998); single- and double-stranded breaks of the sugar-phosphate backbone (Tice and Setlow 1985; Haber 1999; Vilenchik and Knudson 2003); base alkylation (Tice and Setlow 1985; Kadlubar *et al.* 1998; VanderVeen *et al.* 2003); cytosine deamination to uracil (Tice and Setlow 1985; Saparbaev and Zharkov 2017); and deamination of 5-methylcytosine to thymine (Greenblatt *et al.* 1994; Neddermann *et al.* 1996; Sassa *et al.* 2016). Examples of exogenous DNA damage include: ultraviolet (UV) light (Ikehata and Ono 2011); ionizing radiation (Keszenman *et al.* 2015); tobacco (Alexandrov *et al.* 2016); aristolochic acid (Moriya *et al.* 2011); and aflatoxin (Letouzé *et al.* 2017). Mutations are also thought to result from spontaneous ionization or isomerization (i.e. tautomerization) of DNA bases, which can alter base pairing characteristics (Russo *et al.* 1998; Podolyan *et al.* 2000; Masoodi *et al.* 2016; Kimsey *et al.* 2018).

It is important to note that these processes do not affect all bases equally. Each of the 4 nitrogenous bases has its own distinct set of chemically reactive moieties (e.g. amines, carbonyls, or labile ring atoms) (Alberts *et al.* 2014). For any given DNA damaging process or agent, the base(s) with moieties that readily react will be damaged more frequently than bases without such reactive moieties. Local sequence context can also be a key determinant of vulnerability to damage. Mutational signatures are recurrent patterns of base changes that reflect these forms of specificity: the signatures arise naturally because each particular mutagenic process or DNA damaging agent is more likely to affect certain bases in specific contexts more frequently than others (Alexandrov *et al.* 2020).

Mutational signatures typically are inferred using a nonnegative matrix factorization (NMF) algorithm (Alexandrov *et al.* 2013). NMF takes a mutational dataset as input. It initiates by essentially guessing a solution set of constituent signatures with estimated contributions from each putative signature, and then computes the error when attempting to reconstruct the original dataset using that solution set. NMF then tries a slightly different solution set and recomputes the error. This process loops until finding an optimal solution set that stably minimizes reconstruction error. A globally stable solution set is found when different initial conditions all converge to yield that solution set.

NMF analysis can extract reproducible, recurrent patterns of mutations, which often reflect distinct mutagenic processes or DNA damaging agents. There are many mutational signatures with well-established etiologies, including: single base substitution signature 1 (SBS1) from deamination of 5-methylcytosine at CpG motifs; SBS2 and SBS13 from enzymatic deamination of cytosine at TC motifs by APOBEC deaminases; SBS3 from deficiencies in homologous recombination DNA repair; SBS4 and SBS29 from tobacco smoking and chewing habits, respectively; SBS6, SBS15, SBS21, SBS26, and SBS44 from various deficiencies in DNA mismatch repair; SBS7 from UV light exposure; SBS10 from mutation of DNA polymerase epsilon; SBS18 from reactive oxygen species; SBS30 and SBS36 from DNA base excision repair deficiencies; and so forth (Alexandrov *et al.* 2020). About one-third of currently defined mutational signatures remain of unknown etiology (Alexandrov *et al.* 2020).

Previously, the International Agency for Research on Cancer (IARC) named a number of high-priority carcinogens that required further research to fill significant gaps in knowledge (International Agency for Research on Cancer 2010). Among these high-priority carcinogens are 2 small aldehyde compounds, formaldehyde (CH_2_O) and acetaldehyde (C_2_H_4_O). Formaldehyde is classified as a known human carcinogen by IARC, based in part on the evidence of occupational exposure being associated with nasal and nasopharyngeal cancers (International Agency for Research on Cancer 2012a; National Toxicology Program 2014). Formaldehyde is also produced endogenously in cells, as a major metabolic by-product from amino acid metabolism, resulting in high concentrations of up to ∼100 μM in human blood (National Toxicology Program 2014). Acetaldehyde is a reactive compound that humans are commonly exposed to as a result of ethanol consumption, as the initial step of ethanol detoxification is oxidation to acetaldehyde. Like formaldehyde, acetaldehyde is also classified as a known human carcinogen (Secretan *et al.* 2009). Alcohol consumption is associated with higher risk of multiple types of cancer, including: head and neck; esophageal; liver; breast; and colorectal (International Agency for Research on Cancer 2012b). Acetaldehyde associated with alcohol consumption is thought to be causative for cancers of the esophagus and the upper aerodigestive tract (including head and neck), i.e. at sites of highest direct exposure (International Agency for Research on Cancer 2012b).

Understanding the mutagenic characteristics of formaldehyde and acetaldehyde remain important research questions, which can provide valuable insights into the possible roles of these common small aldehydes in cancer mutagenesis and carcinogenesis. Previous attempts to define the mutational patterns induced by formaldehyde and acetaldehyde (e.g. Kucab *et al.* 2019; Dingler *et al.* 2020) have been rather inconclusive, with no demonstrated link to defined mutational signatures in cancers. Here, we report a more detailed understanding of the mutational characteristics of both formaldehyde and acetaldehyde and show that the mutational pattern induced by formaldehyde is similar to a common cancer mutational signature that is currently of unknown etiology, namely single base substitution signature 40.

## Materials and methods

### Reagents and consumables

Bacto peptone (product code 211677) and yeast extract (212750) were purchased from Becton, Dickinson and Co. (Franklin Lakes, NJ). Canavanine (C9758), adenine sulfate dihydrate (AD0028), formaldehyde (F8775), and acetaldehyde (W200344) were purchased from MilliporeSigma (St. Louis, MO). Formaldehyde and acetaldehyde solutions were stored in gas-tight tubes in the dark under nitrogen atmosphere. Agar (FB0010), glucose (GB0219), hygromycin (BS725), PCR purification spin column kit (BS654), agarose (D0012), and Tris-Borate-EDTA buffer (A0026) were purchased from BioBasic (Markham, ON). G418 sulfate (450-130) was purchased from Wisent (St-Bruno, QC). Q5 PCR kits were purchased from New England Biolabs Canada (Whitby, ON). Gas-tight glass tubes with septa (2048-18150) and accessories (2048-11020 and 2048-10020) were purchased from Bellco Glass Inc. (Vineland, NJ).

### Yeast genetics and mutagenesis

Mutagenesis experiments used the ySR127 yeast strain, a *MATα* haploid bearing the *cdc13-1* temperature sensitive allele. In addition, ySR127 has a cassette of 3 reporter genes (*CAN1*, *URA3*, and *ADE2*) near the de novo left telomere of chromosome V. These 3 genes had been deleted from their native loci. Details about ySR127 were described previously (Chan *et al.* 2012) and the strain is available upon request.

Formaldehyde mutagenesis experiments were initiated by inoculating single colonies separately into 5 mL of YPDA rich media (2% Bacto peptone, 1% Bacto yeast extract, 2% glucose, supplemented with 0.001% adenine sulfate) in round bottom glass tubes. Cells were grown at permissive temperature (23°C) for 3 days. Then, cultures were diluted 1:10 into fresh media in gas-tight glass tubes, shifted to restrictive temperature (37°C), and shaken gently at 150 RPM for 3 h, with syringe needles inserted through the septa to enable gas exchange. After a 3-h temperature shift, aliquots of formaldehyde stock solution diluted in media were injected into each tube to obtain the reported final concentrations. Samples were then shaken gently at 150 RPM at 37°C for 3 more hours, in completely sealed gas-tight tubes, to prevent escape of formaldehyde. When formaldehyde treatment was complete, cells were collected by syringe, lightly centrifuged, washed in water, and plated (using a turntable and cell spreader) onto synthetic complete media to assess survival and onto canavanine-containing media with 0.33× adenine to select for mutants (Can^r^ colonies were off-white while Can^r^ Ade^−^ colonies turn red or pink). Care was taken to handle cells gently throughout, as they were quite fragile. Further details of this plating procedure were described in detail previously (Chan 2018).

Acetaldehyde mutagenesis experiments were carried out similarly. We found that we could simplify the acetaldehyde experiments by using tightly sealed 50 mL polypropylene tubes for the temperature shift and mutagen treatment, presumably because acetaldehyde is less volatile than formaldehyde and does not require as fastidious gas-tight containment. Similar results were obtained for acetaldehyde treatment when using either type of tubes. Statistical analyses and data visualizations were done using base R version 4.1 (R Core Team 2020) and tidyverse package version 1.3 (Wickham *et al.* 2019).

### Illumina whole genome sequencing and data analyses

Can^r^ Ade^−^ mutants from formaldehyde and acetaldehyde treatment experiments were collected and reporter gene loss of function phenotypes was verified as described previously (Chan *et al.* 2012). Briefly, Can^r^ red/pink mutants were streaked on YPDA plates. A single colony from each streak was patched onto YPDA. Patches were then replica plated onto glycerol, adenine dropout, canavanine, and uracil dropout media. Mutants that grew on glycerol (i.e. were respiration competent) and Can^r^ Ade^–^ Ura^+^ were considered suitable for sequencing. Can^r^ Ade^−^ Ura^−^ mutants were avoided because those isolates sometimes turn out to be telomere truncations. Mutants from 4, 6, 8, and 10 mM formaldehyde exposure were chosen for sequencing as these had high induced mutation frequencies. For acetaldehyde, mutants from 75 mM treatment were selected for sequencing, as this concentration was most mutagenic. No-aldehyde controls were isolated similarly, except that they were Can^r^ Ade^-^ mutants from 24-h temperature shifts without added mutagen. This longer shift was necessary for controls to acquire more mutations for analysis. Shorter temperature shift without added mutagen would have yielded fewer variants, and sequencing many more control genomes to compensate was not practicable due to budgetary constraints. Twenty-four-hour shifts in the presence of mutagen also were not possible, resulting in very high lethality.

Illumina library preparation and WGS were outsourced to Genome Québec (McGill University, Montréal) or performed on an Illumina MiSeq in our lab. Bowtie2 version 2.3.5.1 (Langmead and Salzberg 2012), SAMtools 1.9 (Li *et al.* 2009), and bcftools 1.9 (Li 2011) were used to map the Illumina reads and call variants. The ySR127 reference sequence was obtained soon after strain construction and represents that genome in an unmutated state, so the variants acquired from each treatment condition can be easily identified. This reference sequence was previously released publicly on NCBI (Chan *et al.* 2015). To map reads to the ySR127 reference and create a sorted BAM file, we ran the following command on each sample: “bowtie2 –local -x ySR127 -1 sample_R1.fastq.gz -2 sample_R2.fastq.gz | samtools view -bS | samtools sort -o sample.bam.” To call variants and output to a BCF file: “bcftools mpileup -Ou -f ySR127.fa sample.bam | bcftools call –ploidy 1 -v -c -Ou -o sample.bcf.” Variants with quality score <30 and/or with sequencing coverage <10 were filtered out: “bcftools view sample.bcf -e ‘INFO/DP < 10’ | bcftools view -e ‘QUAL < 30’ | bcftools view -Ov -o sample.vcf.” VCF file for each sample was then compressed and indexed: “bgzip -c sample.vcf > sample.vcf.gz” and “tabix -p vcf sample.vcf.gz.” Sample VCF files were merged to create a unified VCF: “bcftools merge -m none -Ov -o merge.vcf *.vcf.gz,” where * is a wild card variable for the sample names. In this way, if the same variant is found in multiple samples, they were combined into 1 unique variant. The resulting unified VCF files were passed to MutationalPatterns version 3.6.3 (Blokzijl *et al.* 2018) for further analysis and visualization. Other numerical and statistical analyses, and data visualizations were done using base R version 4.1 (R Core Team 2020) and tidyverse package version 1.3 (Wickham *et al.* 2019).

For trinucleotide frequency correction, the Biostrings package version 2.38.0 (Pagès *et al.* 2022) was used to extract trinucleotide counts for the ySR127 yeast and mm10 mouse reference genomes. Following the convention for reporting mutational signatures, counts for each trinucleotide motif centered on C or T were summed with the counts of their respective reverse complements. The proportion of each trinucleotide was then calculated. To infer the expected pattern in mouse, the frequency of each of the 96 channels of a yeast mutational pattern was multiplied by the ratio of corresponding trinucleotide proportions in mouse vs. in yeast. For example, if a given trinucleotide motif is half as abundant in mouse as in yeast, the corresponding expected frequency of mutations in mouse would be scaled by a factor of 0.5 relative to the observed frequency in yeast data.

## Results

### Formaldehyde- and acetaldehyde-induced mutagenesis

We began by assessing mutagenesis and toxicity induced by the addition of formaldehyde or acetaldehyde. These experiments were done using a haploid yeast strain (ySR127) that forms long regions of subtelomeric single-stranded DNA (ssDNA) when shifted to 37°C due to the *cdc13-1* temperature sensitive point mutation (Garvik *et al.* 1995). At 37°C, the cdc13-1 protein dissociates from telomeres, triggering enzymatic resection of unprotected chromosome ends, which in turn activates the DNA damage checkpoint to arrest cells in G_2_ (Garvik *et al.* 1995). The reporter genes *CAN1*, *ADE2*, and *URA3* had been deleted from their native loci and reintroduced to the left subtelomeric region of chromosome V (Chan *et al.* 2012). This mutagenesis system is very well suited to studying weak mutagens, as ssDNA is more prone to mutation than double-stranded DNA and repair using the complementary strand is not possible. This latter point is an important consideration, since DNA lesions induced by formaldehyde in duplex DNA are potential substrates for nucleotide excision repair (Grogan and Jinks-Robertson 2012). The ssDNA system was used previously to study the mutagenic properties of bisulfite and human APOBEC3G cytidine deaminase (Chan *et al.* 2012); abasic sites (Chan *et al.* 2013); reactive oxygen species (Degtyareva *et al.* 2013); human APOBEC3A and APOBEC3B cytidine deaminases (Chan *et al.* 2015); and alkylating agents (Saini *et al.* 2020).

**Fig. 1.**
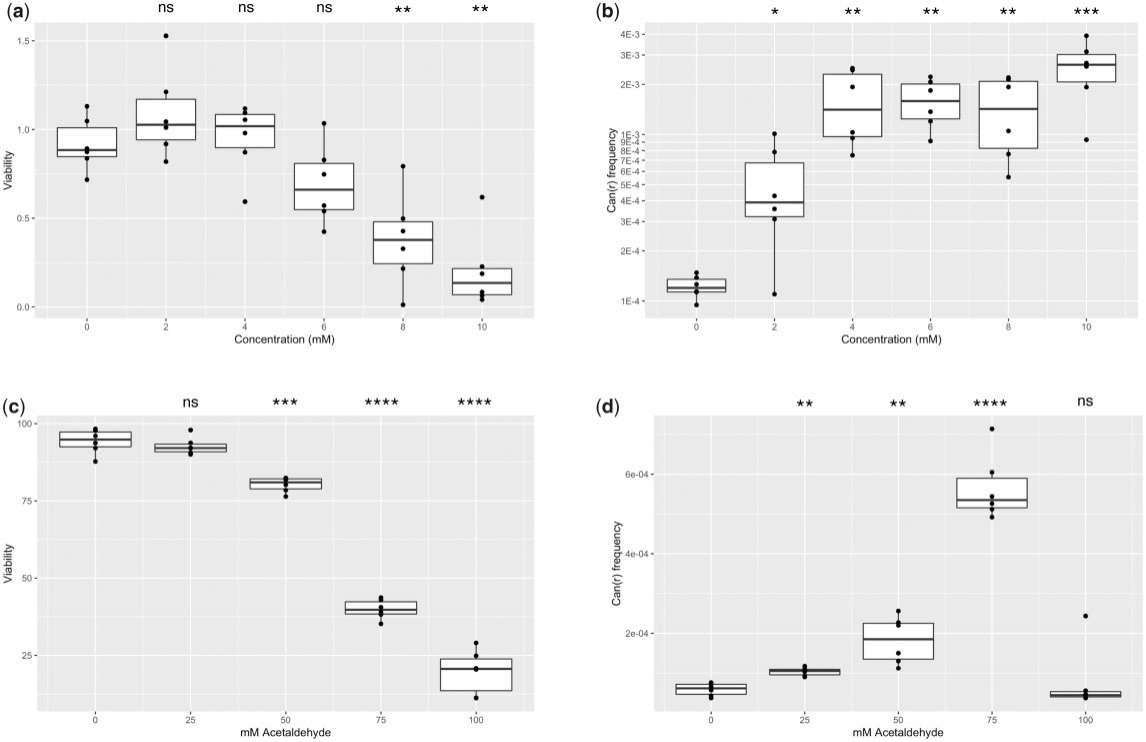
a) Viability and b) *CAN1* inactivation frequency of yeast treated with 0, 2, 4, 6, 8, or 10 mM formaldehyde. c) Viability and d) *CAN1* inactivation frequency of yeast treated with 0, 25, 50, 75, or 100 mM acetaldehyde. Data are from 6 biological replicates for each aldehyde. * denotes *P* < 0.05, ** denotes *P* < 0.01, *** denotes *P* < 0.001, **** denotes *P* < 0.0001, and ns denotes no significant difference by paired *t*-test.

We treated temperature-shifted cells with increasing concentrations of formaldehyde or acetaldehyde. Care was taken to seal the formaldehyde-treated samples in gas-tight tubes; otherwise, the formaldehyde would simply volatilize into the gaseous phase and escape into the atmosphere. Increasing concentrations of formaldehyde resulted in lower viability (see Fig. 1a). While lower concentrations are relatively well tolerated, 8 mM formaldehyde reduced viability below 50%. Formaldehyde-induced inactivation of *CAN1* was detected from as little as 2 mM treatment (median gene inactivation frequency of 3.3 × 10^−4^, see Fig. 1b). Mutagenesis plateaued from 4 to 8 mM formaldehyde, with median mutation frequencies of ∼1.5 × 10^−3^. Mutagenesis peaked at 10 mM formaldehyde exposure (median mutation frequency = 2.7 × 10^−3^), but with a steep decrease in viability. Mock-treated cells (i.e. 0 mM formaldehyde) had median mutation frequency of only 1.2 × 10^−4^. These results show that when the experiments are set up properly to contain the mutagen, formaldehyde is clearly mutagenic to our ssDNA model system.

Cells were considerably more tolerant of higher concentrations of acetaldehyde. We tested concentrations from 25 to 100 mM. Cells treated with lower concentrations (25 and 50 mM) retained high viability, but higher concentrations induced significant lethality (see Fig. 1c). Unlike formaldehyde, the mutagenesis induced by acetaldehyde did not show a plateau. Instead, here was a gradual increase in *CAN1* inactivation frequency when treated with 25 and 50 mM acetaldehyde (see Fig. 1d). Mutation frequency peaked at over 5 × 10^−4^ when cells were treated with 75 mM acetaldehyde. Interestingly, treatment with 100 mM acetaldehyde did not result in detectable mutagenesis while viability was reduced to below 25%. This suggests that the cells which sustained high levels of DNA damage by 100 mM acetaldehyde likely suffered considerable cytotoxic damage as well and did not survive.

### Formaldehyde and acetaldehyde induce an excess of C/G ≥ A/T transversions

We collected mutagenized isolates for Illumina whole genome sequencing to determine what kinds of genetic variants were induced by either formaldehyde (119 genomes) or acetaldehyde (17 genomes) treatment. Total numbers of variants for each sequenced genome and variant calls are reported in Supplementary Tables 2 and 3, respectively. As one would expect, there were mutational hotspots that were mutated recurrently in different samples. Constructing a mutational profile by tallying the number of occurrences (and recurrences) at each site would likely not be a good representation of intrinsic mutational preference, per se. Recurrence could be due to a trinucleotide being susceptible to mutation, but it might also be due to selection effects. Instead, we aggregated data across all samples in each data set and counted mutated motifs: If a treatment does preferentially mutate a trinucleotide motif, multiple instances of that motif at different genomic loci would be mutated. On the other hand, if mutation at a particular instance of a trinucleotide is observed recurrently but there are few other instances of that trinucleotide being mutated at other loci, then selection is quite possible. We adopted our analytical approach to minimize possible distorting effects of selection.

**Fig. 2.**
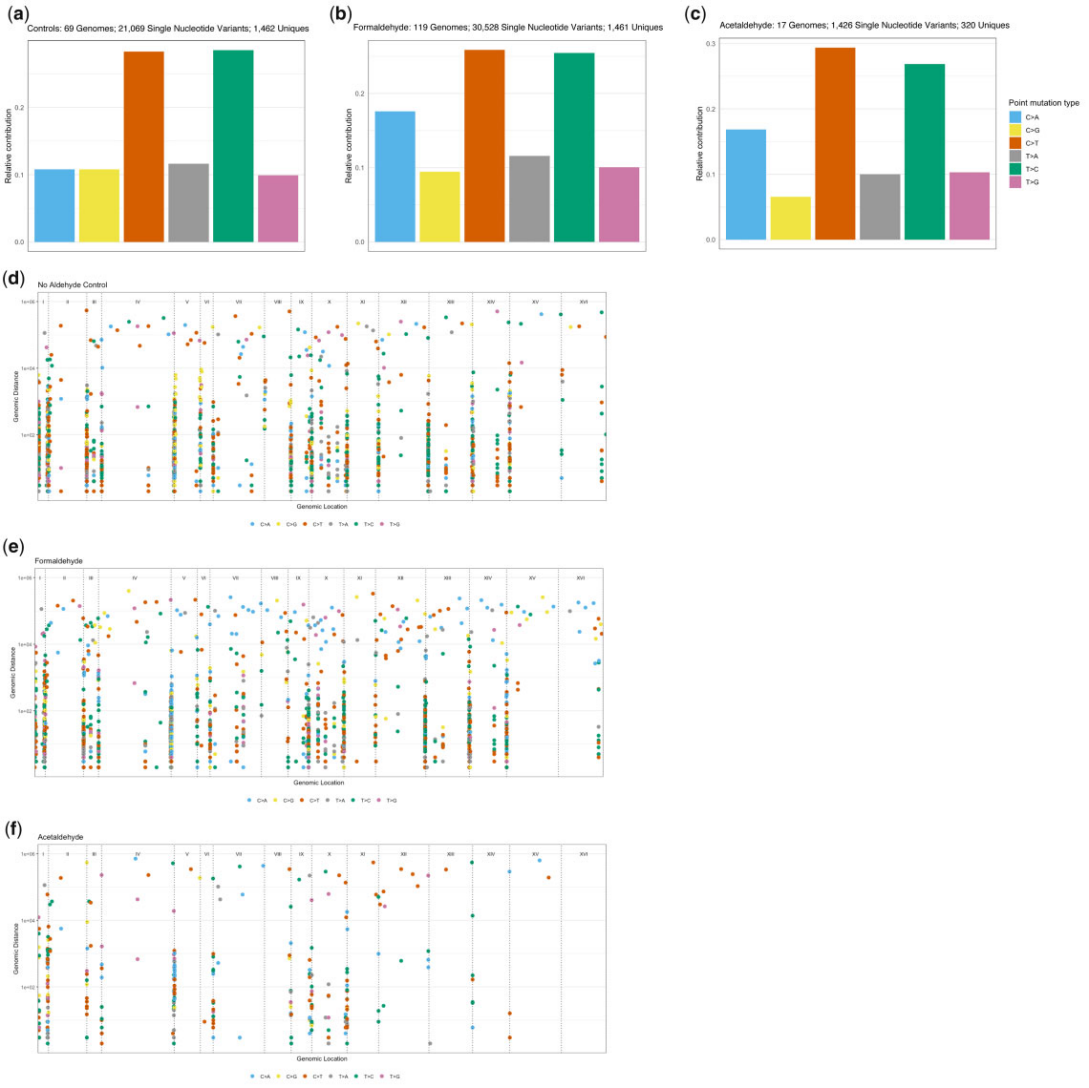
Base substitution types for (a) controls, (b) formaldehyde, and (c) acetaldehyde. Treatment with either aldehyde caused a higher proportion of C/G > A/T transversions. Rainfall plots, showing distance between adjacent mutations, show that most cluster near chromosome ends where ssDNA is enriched, for (d) controls, (e) formaldehyde, and (f) acetaldehyde. Total numbers of sequenced genomes, total numbers of variant calls, and number of unique variants are reported (if the same variant occurs in multiple samples, it is counted as 1 unique).

The genomes mutagenized by either small aldehyde were compared to control genomes that were not treated by either. Analysis of the 69 control genomes revealed a mutational pattern where C/G > T/A and T/A > C/G transitions outnumbered the 4 types of transversions (namely C/G > A/T, C/G > G/C, T/A > A/T, and T/A > G/C, see Fig. 2a), similar to what we had observed previously (Gelova *et al.* 2020). By comparison, formaldehyde and acetaldehyde treatment both caused a relative increase of C/G > A/T transversions (see Fig. 2, b and c). While these substitutions accounted for 11% of the mutational spectrum in untreated controls, this fraction rose to about 17% in the aldehyde-mutagenized genomes. This increase is a common characteristic of mutagenesis caused by small aldehydes in regions of ssDNA.

Since ssDNA should be enriched near the chromosome ends, most variants should map in such regions. To check this, we constructed genome-wide rainfall plots for controls, formaldehyde-, and acetaldehyde-treated isolates (see Fig. 2, d–f, respectively). These graphs show the number of base pairs between adjacent mutations. Consistent with expectation, variants tended to cluster near chromosome ends. Higher-resolution views showing individual chromosomes are available in Supplementary Figs 1, 2, and 3.

### Acetaldehyde induces deletions of 5 or more bases, but formaldehyde does not

**Fig. 3.**
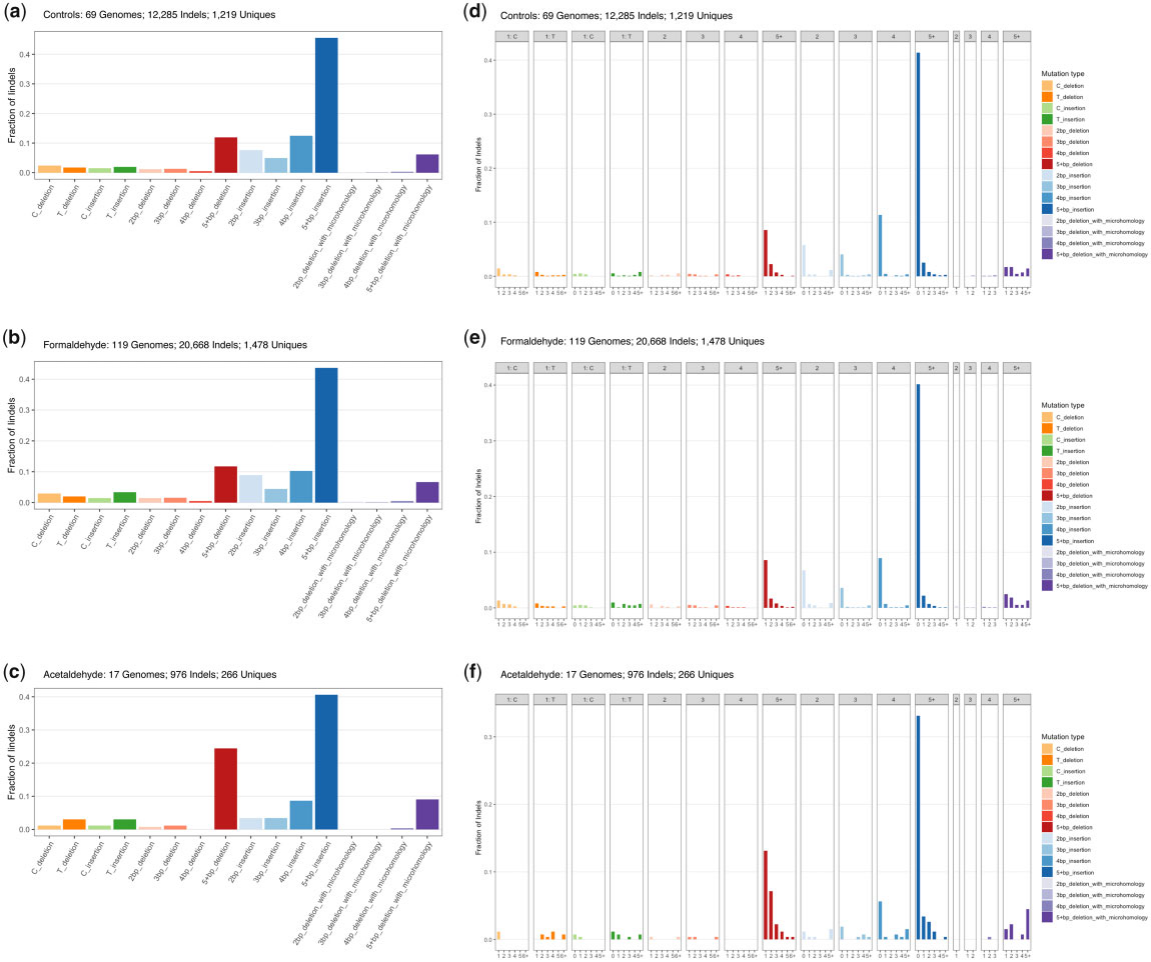
Small indels from (a) no-aldehyde controls, (b) formaldehyde, and (c) acetaldehyde. The different categories comprise: single base deletions or insertions at C/G or T/A base pairs; 2, 3, 4, or 5+ base pair deletions or insertions; and 2, 3, 4, or 5+ base pair deletions with microhomology at break points. Acetaldehyde treatment induces an increased proportion of 5+ base pair deletions (without microhomology). The same small indel data, plotted showing number of repeat units from (d) no-aldehyde controls, (e) formaldehyde, and (f) acetaldehyde. For the single-nucleotide indels, the number of repeat units is the length of a homopolymer run. For indels of dinucleotide, trinucleotide, or greater length, the number of repeat units indicates how many copies of the inserted or deleted unit are immediately adjacent to the site of the indel. Total numbers of sequenced genomes, total numbers of indel calls, and number of unique indels are reported (if the same indel occurs in multiple samples, it is counted as 1 unique).

We also analyzed short insertions and deletions (indels) to determine if treatment with either small aldehyde can induce these genetic changes. The profile of short indels in untreated controls consists mainly of insertions of 5 or more bases, with smaller proportions of shorter insertions as well as deletions of 5 or more bases (see Fig. 3a). The profile of short indels in formaldehyde-mutated genomes is essentially the same as in untreated control genomes, i.e. we did not find evidence that formaldehyde induces a higher proportion of any type of indels (see Fig. 3b). In contrast, there was a notable difference in the acetaldehyde-induced profile of indels: an excess of deletions of 5 or more bases was observed (24% in acetaldehyde vs. 12% in controls, see Fig. 3c). This is a distinguishing property of acetaldehyde-induced DNA damage in the ssDNA system. Plotting these data while grouping by number of repeat units adjacent to each indel confirmed the excess of these deletions from acetaldehyde treatment (compare Fig. 3, d–f). The most frequent events were deletion of a single unit. Deletions were less frequent as the number of repeat units increased, likely because longer tandem sets of repeats were simply more rare.

### Formaldehyde and acetaldehyde produce distinct mutational patterns

**Fig. 4.**
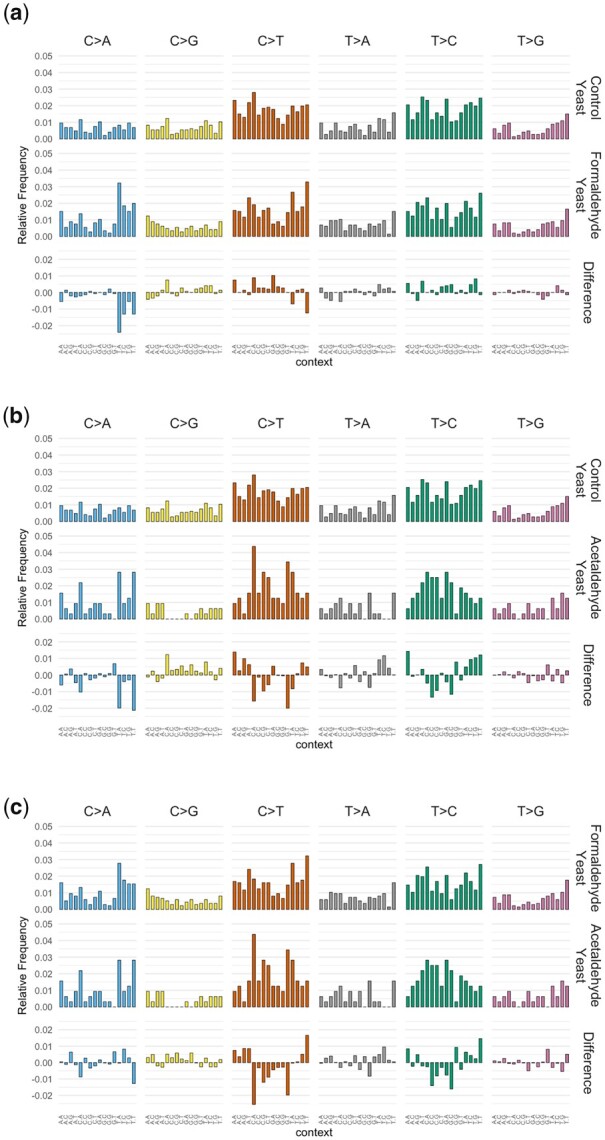
Comparisons of mutational patterns between (a) controls and formaldehyde; (b) controls and acetaldehyde; and (c) formaldehyde and acetaldehyde.

To investigate the mutational properties of the small aldehydes in more detail, we plotted their mutational profiles in the 96-channel format of the COSMIC mutational signatures. By this convention, all substitutions are reported as originating from a pyrimidine base, i.e. same as the mutation spectra reported above. In addition, the 96-channel profiling features trinucleotide motifs consisting of the mutated base, flanked by an adjacent base 5ʹ and 3ʹ. Cosine similarity is a metric for comparing mutational patterns, yielding a maximum value of exactly 1 for 2 identical patterns (Alexandrov *et al.* 2013). The mutational pattern of formaldehyde is similar to untreated controls (cosine similarity = 0.93), but the excess of C/G > A/T transversions is nonetheless evident (see Fig. 4a). The mutational pattern of acetaldehyde is more dissimilar vs. the profile of untreated controls (cosine similarity = 0.868), but again with a noticeable excess of C/G > A/T substitutions (see Fig. 4b). When comparing the formaldehyde and acetaldehyde profiles directly to one another, the cosine similarity value is 0.882, showing some similarities but also clear differences in the C/G > T/A channels especially (see Fig. 4c).

**Fig. 5.**
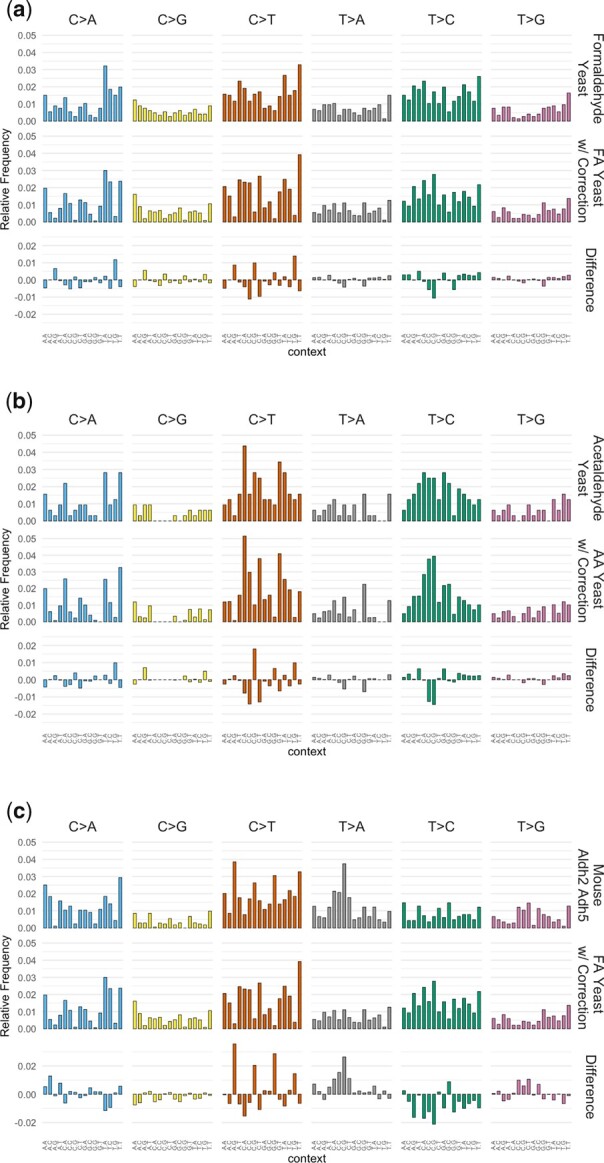
Comparisons of mutational patterns between (a) formaldehyde with and without correction for trinucleotide frequencies in mouse; (b) acetaldehyde with and without correction for trinucleotide frequencies in mouse; and (c) Aldh2 Adh5-deficient mouse cells and trinucleotide frequency corrected yeast treated with formaldehyde.

A recent study described mutational patterns obtained in mice that were genetically deleted for genes important in aldehyde detoxification, ADH5 and ALDH2, thus leading to buildup of endogenous aldehydes (Dingler *et al.* 2020). To compare our mutational patterns derived from mutagenized yeast genomes to these profiles from mice, we first adjusted for differences in trinucleotide abundances between the 2 species to obtained corrected mutational patterns (see Fig. 5, a and b). Applying this adjustment is necessary to obtain the corrected mutational pattern for a more accurate comparison between species. A main difference between the yeast and mouse genomes is the lower abundance of CpG motifs in the latter. Nonetheless, the corrected mutational patterns retained high similarity to the original (uncorrected) patterns in yeast (cosine similarity values > 0.95).

When we compared the various mutational patterns, we noticed that cosine similarity values are relatively low when comparing between the corrected yeast patterns we derived and the mouse patterns from Dingler *et al.* (2020) (see Table 1). These values are somewhat higher when comparing the formaldehyde pattern in yeast to the mouse patterns. A closer examination of these profiles from mouse suggests that there are likely to be mutations from other sources mixed in the mouse patterns, e.g. from SBS1 (deamination of 5-methylcytosine at CpG motifs, see Fig. 5c). We also noted some differences among the various mouse patterns themselves: while the ones from Adh5^−/−^ and Aldh2^−/−^ Adh5^−/−^ had cosine similarity = 0.887, the Aldh2^−/−^ profile was noticeably more dissimilar (cosine similarity <0.8 vs. the other 2 profiles, see Table 2). This is consistent with the likelihood that there are other mutation sources mixed in with the mouse mutational patterns, which may be confounding interpretation of a hypothesized pattern induced by excess endogenous aldehydes.

**Table 1.** Cosine similarity values between mutational profiles of (FA) formaldehyde- and (AA) acetaldehyde-mutagenized yeast (with correction for trinucleotide frequencies in mouse) and of mice deficient for aldehyde detoxification genes from (Dingler *et al.* 2020).

	Mouse Aldh2^−/−^	Mouse Adh5^−/−^	Mouse Aldh2^−/−^ Adh5^−/−^
Yeast FA, corrected	0.658	0.735	0.767
Yeast AA, corrected	0.617	0.633	0.673

**Table 2.** Cosine similarity values among mice deficient for aldehyde detoxification genes from (Dingler *et al.* 2020).

	Mouse WT	Mouse Aldh2^−/−^	Mouse Adh5^−/−^	Mouse Aldh2^−/−^ Adh5^−/−^
Mouse WT	1	0.832	0.845	0.838
Mouse Aldh2^−/−^		1	0.780	0.774
Mouse Adh5^−/−^			1	0.887
Mouse Aldh2^−/−^ Adh5^−/−^				1

### Formaldehyde mutational pattern resembles COSMIC SBS signature 40

**Fig. 6.**
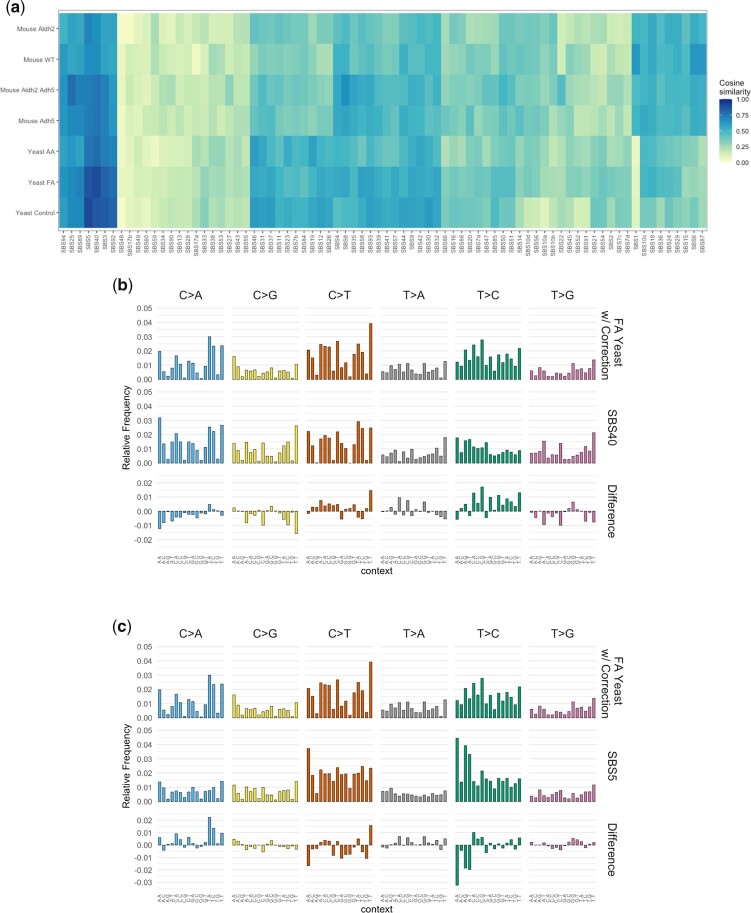
a) Cosine similarity heatmap with hierarchical clustering comparing COSMIC SBS signatures vs. mouse mutational patterns from Dingler *et al.*; and vs. trinucleotide abundance-corrected mutational patterns in yeast from acetaldehyde, formaldehyde, and no-aldehyde control. b) Comparison of corrected formaldehyde mutational pattern in yeast vs. SBS signature 40. c) Comparison of corrected formaldehyde mutational pattern in yeast vs. SBS signature 5.

We then investigated whether these mutational patterns might shed light on the etiology of any known COSMIC mutational signatures. We started with the mouse profiles published by Dingler *et al.* (2020) and confirmed that none of the mouse profiles showed a particularly close resemblance to any known COSMIC signature (see Fig. 6a and Supplementary Table 4). All of those cosine similarity values were <0.8, suggesting that if there are bona fide COSMIC signatures within the mouse mutational patterns, they are possibly obscured by being in a mixture of multiple signatures.

Comparison of the corrected acetaldehyde pattern from yeast vs. known COSMIC signatures also yielded, at best, cosine similarity of 0.79 to SBS40, a signature of unknown etiology (see Fig. 6a and Supplementary Table 4). Since we had better direct control of the induced mutagenesis experiments using the yeast system with exogenously applied mutagen, it does not seem as likely that other mutagenic processes are obscuring the acetaldehyde-induced pattern. We conclude that the acetaldehyde pattern we obtained is not a plausible match for any known COSMIC signature at this point.

Finally, we compared the formaldehyde pattern to the COSMIC signatures, finding that the closest match is to SBS40, with cosine similarity = 0.9 (see Fig. 6, a and b). The second closest match was to SBS5 (cosine similarity = 0.864, see Fig. 6, a and c). We previously studied an SBS5-like mutational pattern in yeast and showed that similar patterns are widely conserved in many species. The no-aldehyde control mutational pattern was indeed SBS5-like (cosine similarity = 0.907, see Fig. 6a and Supplementary Table 4). Moreover, the SBS5-like pattern is due to error-prone translesion DNA synthesis in the absence of added mutagens and increases with increasing sugar metabolism (Gelova *et al.* 2020). An SBS40-like mutational pattern would require a separate explanation, which would be the addition of exogenous formaldehyde to our experimental system. As such, we propose that a plausible etiology for SBS40 in cancers is the mutagenicity of formaldehyde.

## Discussion

In this article, we report the use of a sensitive ssDNA-based mutagenesis reporter system to characterize the mutagenic properties of 2 small aldehydes, formaldehyde and acetaldehyde. This system is especially well suited for investigating chemical agents with relatively weak mutagenicity. A challenge of using conventional mutagenesis systems to study weak mutagens is that induced mutations can be rare and it can be difficult to discern a reliable mutational pattern using relatively few mutations (Kucab *et al.* 2019; Dingler *et al.* 2020). In addition to being a more sensitive reporter system, it is considerably more cost-efficient to sequence compact yeast genomes (each ∼12 Mb) than mammalian genomes, which are much larger (∼3 Gb). By applying a correction to account for different abundances of trinucleotide motifs, we can use data from the sequencing of mutagenized yeast to infer the expected mutational pattern in another species. Another key advantage is that the single-stranded configuration of the DNA precludes repair processes requiring a complementary strand. By sidestepping intervention from DNA repair processes, the ssDNA system can provide, in effect, a purer readout of the effects of mutagenesis per se. Leveraging these advantages of the ssDNA mutagenesis reporter system, we were able to infer the mutational patterns of both formaldehyde and acetaldehyde. When conventional systems for studying mutagenesis do not yield clear-cut results, an ssDNA-enriched assay system can be a useful complementary approach.

It is also important to acknowledge the limitations of this system. First, the initial identification of isolates of interest requires selection for reporter gene inactivation. This selection will necessarily reveal recurrent mutational hotspot mutations when isolates are sequenced (Rogozin and Pavlov 2003). To avoid bias to a mutational pattern due to selection, it is possible to filter out variant calls that map to the reporter genes, although this could mean discarding a significant fraction of variants. Alternatively, it is possible to essentially count mutated motifs: if a mutagen does preferentially mutate a given trinucleotide, then multiple instances of that trinucleotide would be mutated at different genomic loci, as opposed to a recurrent hotspot due to selection. Another limitation is the haploidy of the system. While this facilitates identification of isolates enriched for ssDNA exposure, there is a tradeoff that haploids are not as buffered against potentially deleterious variants as diploids.

The 2 small aldehydes share some similar mutagenicity characteristics but also have their differences. Both induce dose-dependent increases in mutagenesis at lower concentrations. But whereas formaldehyde-induced mutagenesis essentially plateaus from 4 mM up to 8 mM, acetaldehyde-induced mutagenesis peaks at 75 mM and then drops sharply at the even higher concentration of 100 mM. Both aldehydes induce significant cytotoxicity at the higher end of their respective ranges of tested concentrations, but yeast are able to tolerate considerably higher doses of acetaldehyde overall. Yeast are presumably evolved to cope with significantly higher concentrations of acetaldehyde, since it is an abundant intermediate in ethanol production from fermentation (Matsufuji *et al.* 2008). Both aldehydes cause an excess of C/G > A/T transversions, which is consistent with previous reports showing preferential adduct formation and mutagenesis at guanines (Crosby *et al.* 1988; Ohta *et al.* 1999; Yasui *et al.* 2001; Liu *et al.* 2006; Stein *et al.* 2006; Upton *et al.* 2006a,b). Interestingly, acetaldehyde induces an excess of deletion variants of 5 or more bases in our system, but formaldehyde does not, consistent with previous reports (Yasui *et al.* 2001; Garaycoechea *et al.* 2018). These various mutagenic characteristics of formaldehyde and acetaldehyde reflect their chemical similarities and differences. A limitation of this study is that relatively few acetaldehyde-mutagenized genomes were sequenced, due to budgetary constraints. Despite this, the considerations just discussed lend credence to overall validity of the findings.

The 96-channel mutational patterns of formaldehyde and acetaldehyde revealed further differences between the 2 compounds. Whereas the acetaldehyde pattern did not particularly resemble any known COSMIC signature, new mutational signatures will be revealed as more cancer samples are sequenced and analyzed. Since alcohol consumption is associated with multiple cancer types and it is thought that the acetaldehyde from alcohol detoxification would surely damage DNA (International Agency for Research on Cancer 2012b), associated mutational signature(s) may yet be discovered in the future. The formaldehyde pattern we obtained was similar to SBS signature 40. SBS40 is currently of unknown etiology, but it is known to be present in at least 28 cancer types (Alexandrov *et al.* 2020), making SBS40 the third most common mutational signature in cancers. The high prevalence of SBS40 hints at an endogenous origin for the underlying DNA damage that is present in different cell types throughout the body. Since formaldehyde is produced endogenously and exists at steady-state concentrations in humans in the range of tens of micromolar (National Toxicology Program 2014), it would fit this profile. When all of the available information is taken into consideration, mutagenesis from endogenously generated formaldehyde emerges as a plausible candidate for the etiology of SBS40.

Comparison with mutational patterns from mice deleted for aldehyde detoxification genes suggest that those profiles are likely mixtures of mutations from different mutagenic processes, and not just from DNA damage due to accumulation of excess endogenous aldehydes. For example, the contribution from SBS1 (C/G > T/A at CpG motifs) was quite noticeable. Mutagenesis from other sources likely interferes with making an accurate inference of the aldehyde-associated mutagenesis. This is perhaps another significant challenge when using systems for mutational detection that are not (and maybe cannot) be properly controlled to factor out mutagenesis from other sources. Deployment of more specialized and sensitive mutagenesis detection systems where the experimenters have more direct control over the mutation induction can continue to play an important role in shining new light on mutagenesis.

## Supplementary Material

jkac238_Supplemental_Figures

jkac238_Supplemental_Table_1

jkac238_Supplemental_Table_2

jkac238_Supplemental_Table_3

jkac238_Supplemental_Table_4

## Data Availability

Sequencing reads were uploaded to the NCBI SRA (National Center for Biotechnology Information Sequence Read Archive), accessions PRJNA839792 and PRJNA574140. Details on each sequencing sample are listed in Supplementary Table 1. The ySR127 reference genome is available on NCBI Assembly (accession GCA_001051215.1). Supplemental material is available at G3 online.
