# Supplementary material for: Analyses of mutational patterns induced by formaldehyde and acetaldehyde reveal similarity to a common mutational signature
Source: G3 (Bethesda). 2022 Sep 8;12(11):jkac238. doi: 10.1093/g3journal/jkac238 (PMC9635668; doi:10.1093/g3journal/jkac238)
Supplement: jkac238_Supplemental_Figures [file jkac238_supplemental_figures.pdf]

Supplementary Figure 1: Rainfall plots for each chromosome, drawn to the same horizontal scale (representing lengths of chromosomes), showing single nucleotide variants from controls.

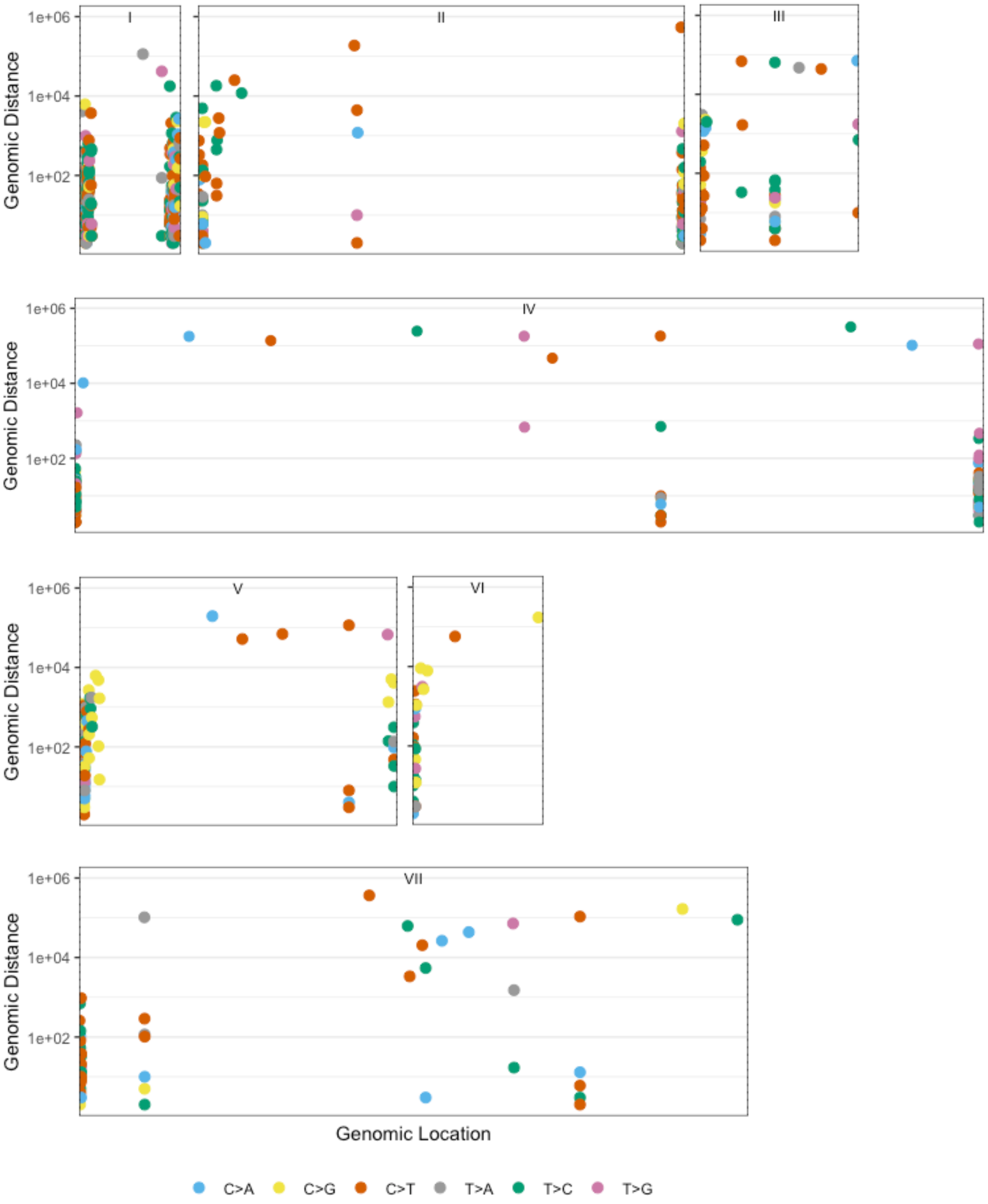

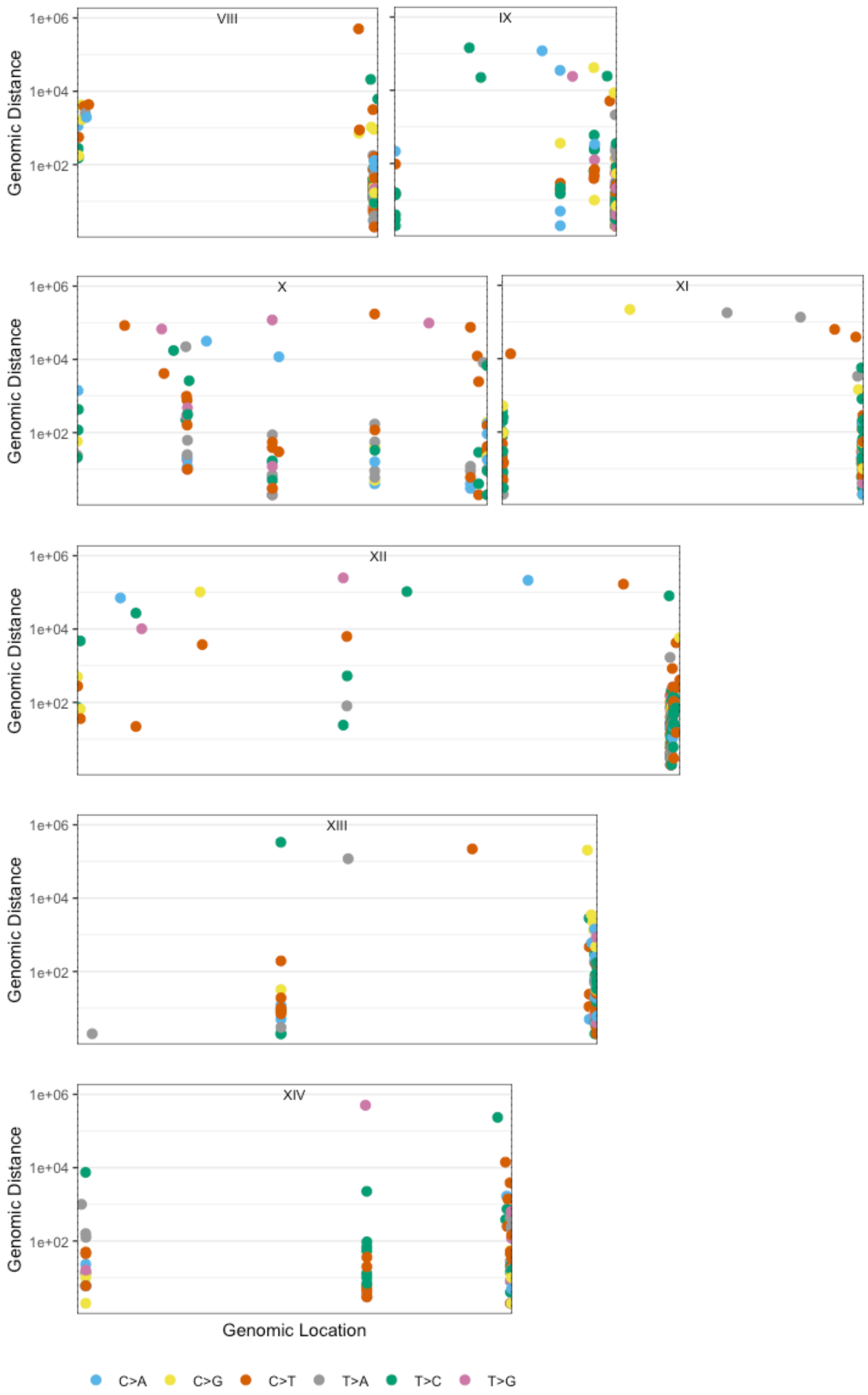

VIII
IX
X
XI
XII
XIII
XIV
1e+06
1e+04
1e+02
Genomic Distance
Genomic Location
C>A
C>G
C>T
T>A
T>C
T>G

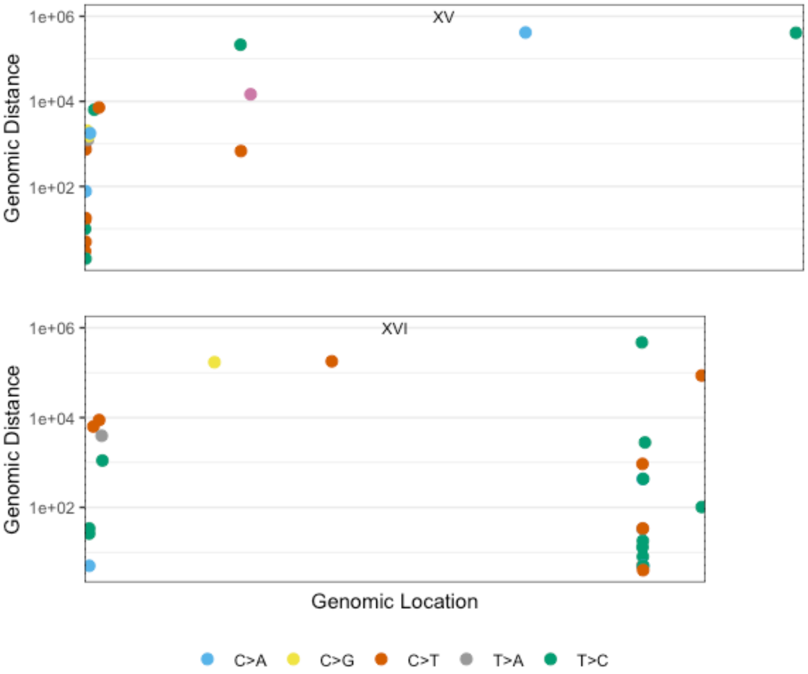
XV
1e+06
1e+04
1e+02
Genomic Distance
XVI
1e+06
1e+04
1e+02
Genomic Distance
Genomic Location
C>A
C>G
C>T
T>A
T>C

Supplementary Figure 2: Rainfall plots for each chromosome, drawn to the same horizontal scale (representing lengths of chromosomes), showing single nucleotide variants from formaldehyde treatment.

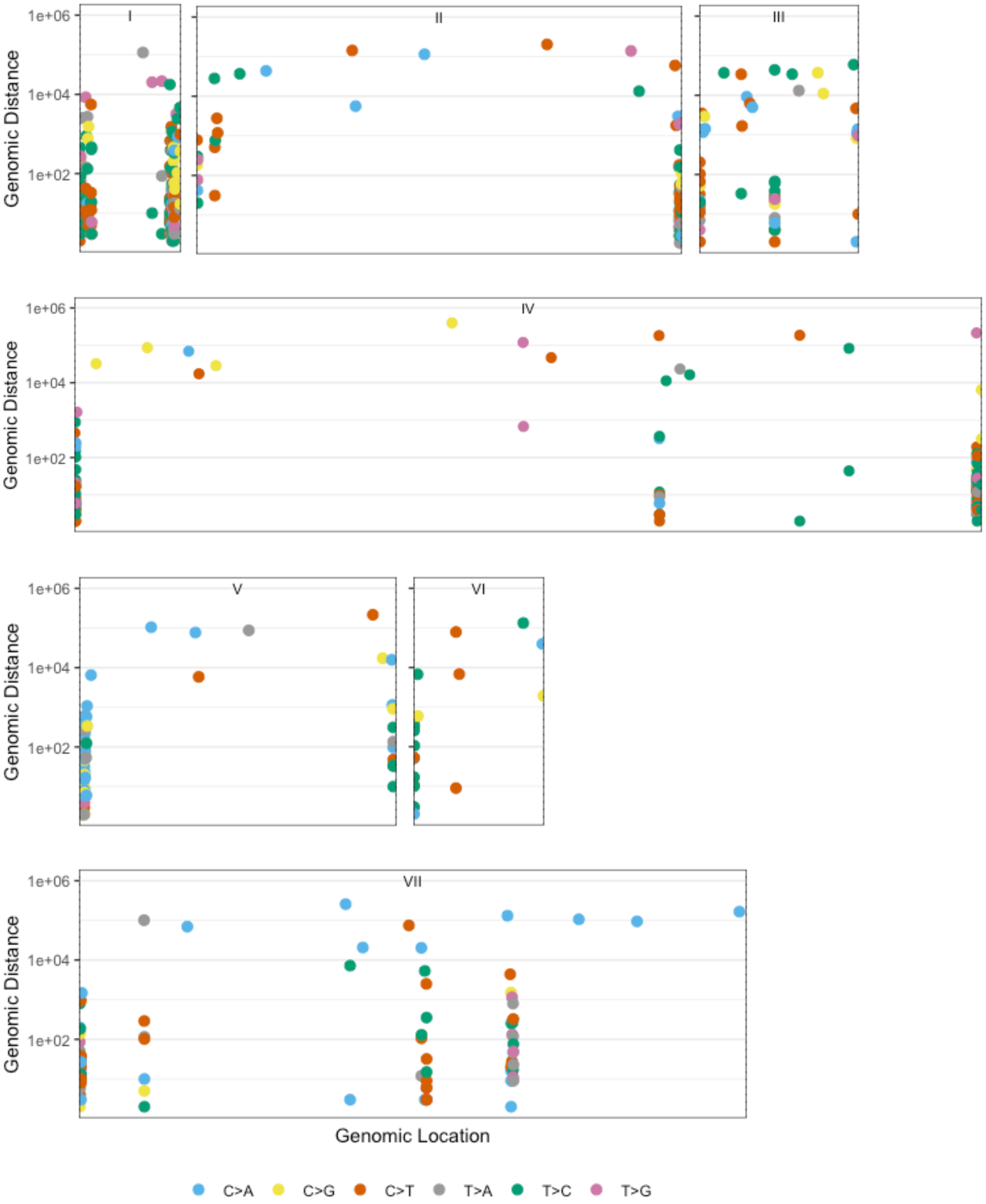

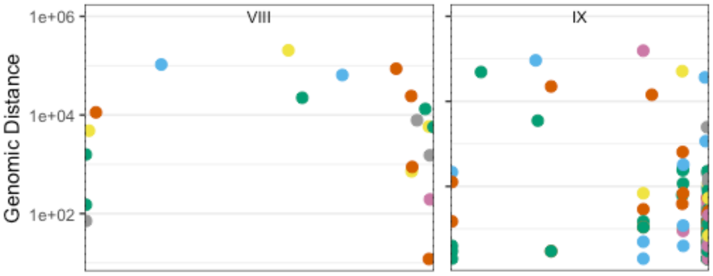
1e+06
1e+04
1e+02
Genomic Distance
VIII
IX

1

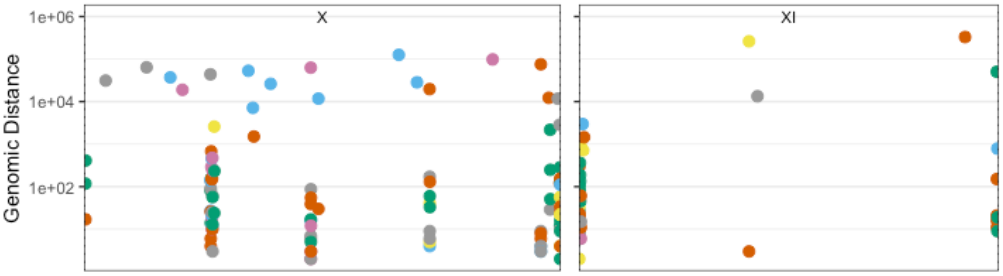
1e+06
1e+04
1e+02
Genomic Distance
X
XI

2

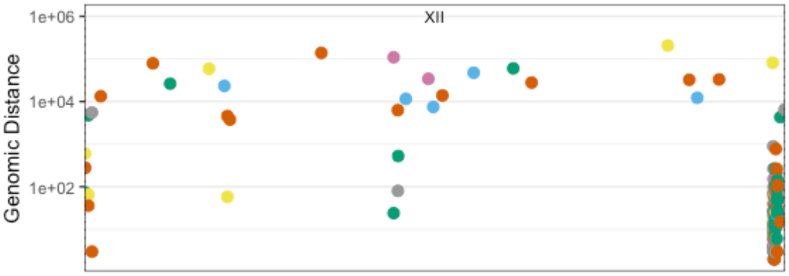
1e+06
1e+04
1e+02
Genomic Distance
XII

3

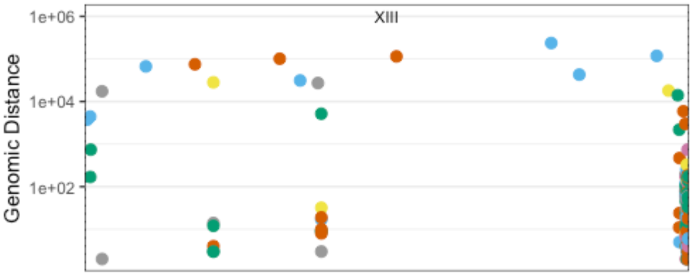
1e+06
1e+04
1e+02
Genomic Distance
XIII

4

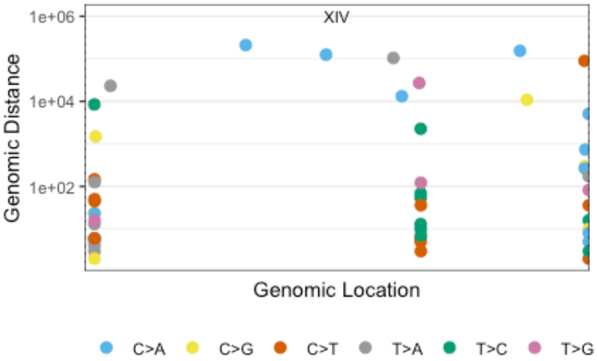
1e+06
1e+04
1e+02
Genomic Distance
XIV
Genomic Location
C>A
C>G
C>T
T>A
T>C
T>G

5

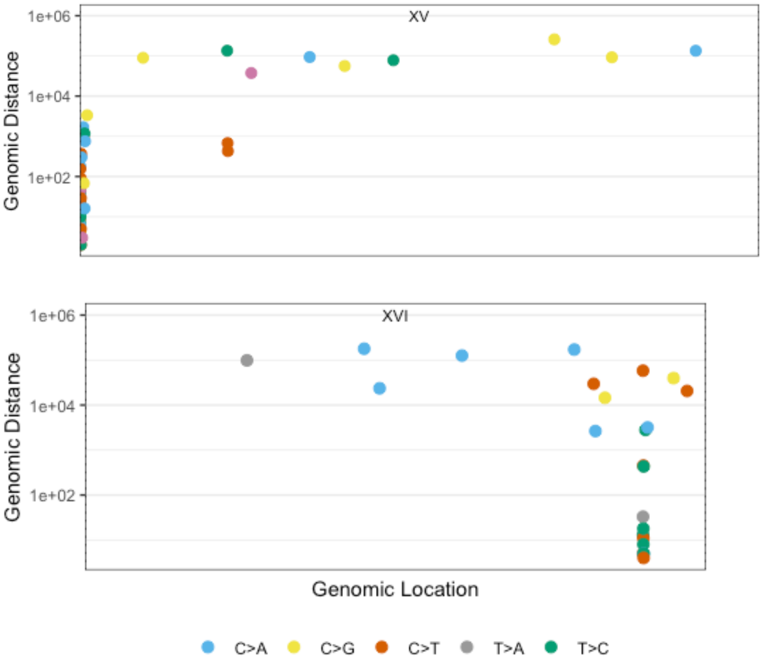
XV
Genomic Distance
1e+06
1e+04
1e+02
XVI
Genomic Distance
1e+06
1e+04
1e+02
Genomic Location
C>A
C>G
C>T
T>A
T>C

Supplementary Figure 3: Rainfall plots for each chromosome, drawn to the same horizontal scale (representing lengths of chromosomes), showing single nucleotide variants from formaldehyde treatment. Chromosome XVI had no variants.

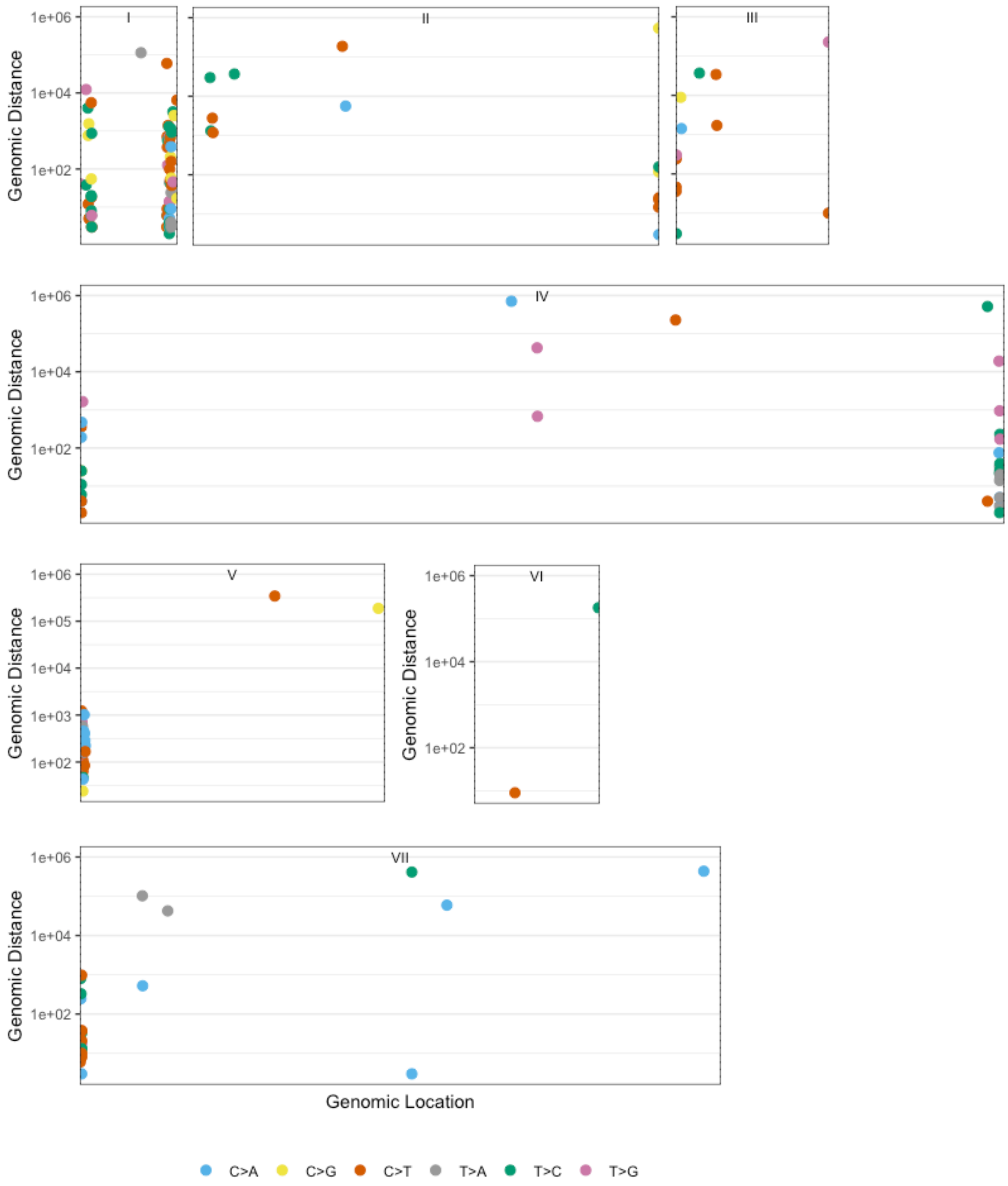

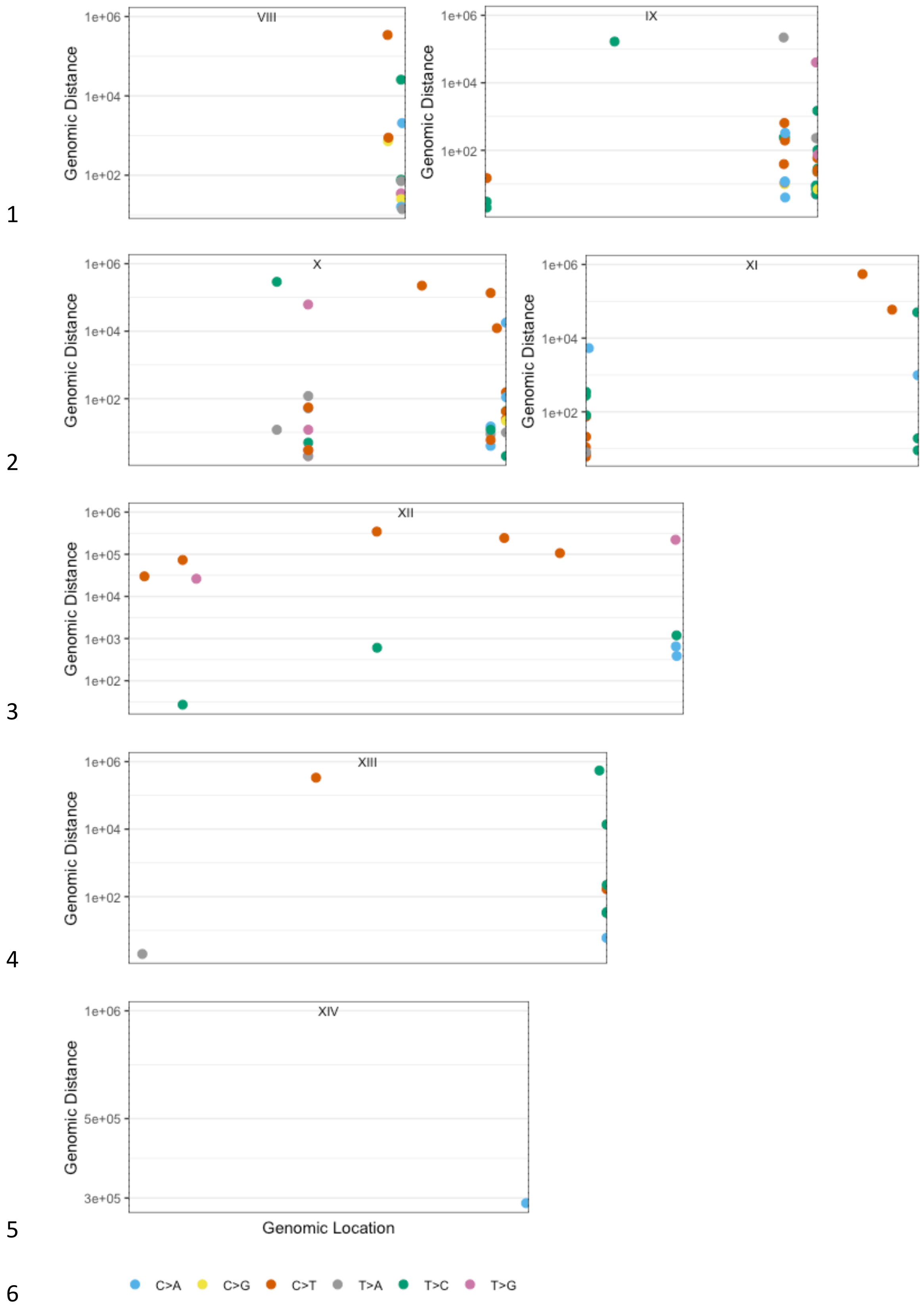
1
2
3
4
5
6
VIII
IX
X
XI
XII
XIII
XIV
Genomic Distance
Genomic Location
1e+06
1e+05
1e+04
1e+03
1e+02
5e+05
3e+05
C>A
C>G
C>T
T>A
T>C
T>G

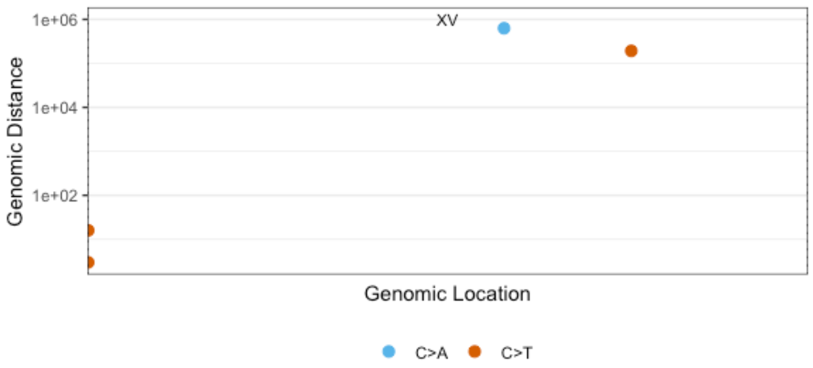
1e+06
1e+04
1e+02
Genomic Distance
XV
Genomic Location
C>A
C>T
